# Supplementary material for: Learning spatial hearing via innate mechanisms
Source: PLoS Comput Biol. 2025 Oct 10;21(10):e1013543. doi: 10.1371/journal.pcbi.1013543 (PMC12668631; doi:10.1371/journal.pcbi.1013543)
Supplement: Attachment — Submitted filename: PLoSCB_ChuEtAl_Review25.pdf [file pcbi.1013543.s001.pdf]

Summary:

The authors address the problem of how accurate auditory spatial maps—specifically for broadband white noise stimuli—can be learned from coarse-grained supervisory signals. They investigate the hypothesis that innate “teacher” circuits in the brain may provide minimal feedback signals that guide the learning of “student” circuits through a process termed *bootstrap learning*. The paper explores several biologically plausible mechanisms by which such teacher circuits might be instantiated, including left/right comparators, midline detectors, and temporal comparators. The authors demonstrate that student networks can learn precise spatial maps, adapt to disruptions in the teacher circuit, and succeed regardless of the specific implementation of the teacher network.

Main review:

The motivation of the paper is clear, and the general problem is of broad interest. The core idea presented in the paper is simple, neat, and has the potential to be expanded more generally.

Having said that, this manuscript can benefit from several clarifications and improvement before being considered for publication. I’m listing my concerns below, and I hope that the authors can clarify them.

1. **How are the authors defining “calibration”:** In several places in the manuscript the concept of “calibration” is referenced without proper definition. Are they referring to the statistical notion of calibration in the sense that uncertainty (or predicted error) in estimation of new observations inversely mirrors accuracy? It seems like here calibration refers to a process akin to fine-tuning. It would be good to define this term operationally since its used in several places in the text.
    a. L6: “adjusting to developmental changes like head growth”: This is a tricky example as its not clear if accuracy is held constant while these changes happen.

2. **Independence of Teacher and Student modules**: Conceptually, in this approach the authors suppose the independence of these two components. In other words, one can swap out different teacher modules, such as the LSO circuitry for other mechanistic implementations, and re-learn the spatial map. But this seems to be a strong constraint in the sense that in biological systems brainstem circuits like the LSO and MSO are early, upstream components of the auditory pathways used for sound localization. This, I believe, will have consequences for the learning process.

3. **System identification under variable source spectra**: A fundamental challenge in sound localization is that listeners typically have no access to the source spectrum, which complicates the inference of spatial location from sensory input. This issue is particularly pronounced for localization in elevation, where spectral cues are more ambiguous. While the paper focuses primarily on azimuthal localization, it remains

unclear how the proposed bootstrapping process would perform—either theoretically or empirically—when the teacher circuit's response does not uniquely map to location (i.e., the response function of the teacher isn't one-one). This ambiguity could undermine the reliability of the supervisory signal and seems critical to address. I recommend the authors provide additional theoretical justification or empirical evidence to support the viability of the approach under such conditions.

4. **LSO teacher implementation**: The performance reported in Fig. 2G doesn't match the authors characterization of "lowest error near the midline and increased errors at ±90". Can the authors replot the these graphs only for this range of azimuths and provide some statistics to back this claim? I'm also unclear on how I must interpret the significant non-monotonicity in these charts.
    a. I'm assuming these errors are single-shot predictions during the test phase. Is this correct?
    b. Can the authors also provide some quantification for how error varies with the number of head movements the agent performs? In other words, in one-shot the agent can estimate if it overshoots or undershoots the target. But what's the sequence of actions that follow after? Do the authors cap the movement to n=2 (as suggested by Algorithm 1)?

5. **Midline detector as intrinsic reward**: Fig. 5 gives the impression that the reward is +1 for the midline and -1 otherwise. While I can understand how this would work if the student has access to the firing rate of the teacher (as it has a smooth drop off on either side that can provide gradient information), it's unclear to me how this binary reward function helps learning. Does the student rely on random exploration of lateral movements until it happens upon the midline? If so, this seems inefficient and potentially implausible. Can the authors clarify?
    a. On the other hand, Fig. 5D (error vs. azimuth) looks quite human-like.

6. **Adaptation vs degradation**: The authors repeatedly emphasize that their approach supports adaptation when aspects of innate function—such as teacher responses—change. However, this could be conceptually distinct from scenarios involving impaired hearing or reduced acuity. In such cases, the "teacher" circuit may remain intact, while the "student" circuit exhibits high localization errors. It is unclear how the proposed bootstrapping mechanism would handle this type of asymmetry. I would appreciate further clarification or intuition in this regard.

7. The authors don't make explicit how they theorize the multiple mechanisms to interact with each other as suggested in the abstract.

8. **Grammatical errors:** There are several places in the manuscript that can benefit from proofreading and editing. I'm listing a few (not exhaustive) below but please do ensure a thorough review.
    a. Abstract: "or other supervisory signal".

b. L92 “ The agent uses Student network”
c. L97 “listen again”
d. L109 “that for a”
e. Fig 1 caption “map need to”
f. Fig 6 heading “unllateral”

9. L423 and L547: sound source localization (SSL) – This acronym isn’t used anywhere else in the text and it also bears the risk of being confused with the more popular “self supervised learning” usage in machine learning.
