# Supplementary material for: Learning spatial hearing via innate mechanisms
Source: PLoS Comput Biol. 2025 Oct 10;21(10):e1013543. doi: 10.1371/journal.pcbi.1013543 (PMC12668631; doi:10.1371/journal.pcbi.1013543)
Supplement: Attachment — Submitted filename: Response to reviewers.pdf [file pcbi.1013543.s002.pdf]

Response to reviews

We thank the editor and reviewers for their comments. Below, we reply to the reviewers' comments. Our replies are in purple.

Reviewer #1

Summary

The manuscript proposes models for the calibration of auditory space without the use of visual cues (or alternative supervisory signals). Evidence is presented that this can be achieved using approximate feedback from a simple innate circuit able to distinguish left from right sound sources. Furthermore, supervised learning can be of benefit to maintain the adaptive neural representation. A number of possible mechanisms are discussed, which may interact in order to maintain an accurate auditory spatial map.

Main comments
The manuscript addresses the question of how humans calibrate auditory spatial hearing, in the absence of a supervisory signal such as vision. This is an important topic in the area of auditory spatial cognition, for which a number of alternative explanations have been proposed, but a satisfactory answer has not yet been established. The paper investigates "bootstrap learning" as a possible process for calibrating the auditory spatial map. Interesting and enjoyable to read, the ideas presented in the paper are intriguing, the methods appropriate, and the interpretation of the results is sound. The manuscript will be of interest to readers of PLOS Computational Biology. I have some comments that the authors may wish to consider, that mostly regard providing additional information and clarification. These are detailed below, which I hope will be of use to the authors.

Introduction: The Introduction is fairly short overall, and some additional information and background may help set the scene for the reader. Movement is a crucial element of the calibration process, as is made clear later on in the manuscript e.g. in Fig 2, (A) Interactive learning procedure with the left/right Teacher circuit. However, the text in the Introduction is not particularly clear regarding this. It is briefly mentioned on line 43 that "The "Agent"–defined here as any human, animal, or model capable of acting within the environment–receives auditory inputs and moves in the simulation," but perhaps more emphasis could be placed on this aspect. The text appears to be discussing the role of audiomotor feedback to calibrate auditory space (e.g. as per Lewald, 2013, who defines audiomotor feedback as the "evaluation of systematic changes of auditory spatial cues resulting from head and body movements)", but it is not entirely clear if this is the case, and it would help if the notion of audiomotor feedback was put into context with regards to the current work in the Introduction.
Lewald, J. (2013). Exceptional ability of blind humans to hear sound motion: implications for the emergence of auditory space. Neuropsychologia, 51(1), 181-186.

Our bootstrap learning proposal is slightly more general, but in this case yes, it's one way of implementing the concept of calibration by audiomotor feedback. We have added the following text to the introduction to make this clearer: "This is one way of implementing the hypothesis of calibration by audiomotor feedback (Bernard et al. 2012, Lewald 2013)". (line 54)

Regarding the question on line 9 ("What could be the calibration signal used by the brain to learn spatial hearing?"), some brief further information about this topic that has been discussed in the

literature might strengthen the background for the reader. For example, calibration of auditory space might result from experience with how auditory spatial cues change with self-motion such as when approaching a sound or turning the head, or using audiomotor feedback from reaching out and touching sound producing objects (Kolarik et al., 2025), and it has been highlighted that information from alternative senses such as touch can help calibrate auditory space during development (Gori et al., 2014 – ref 36 in the manuscript).
Kolarik, A. J., & Moore, B. C. (2025). Principles Governing the Effects of Sensory Loss on Human Abilities: An Integrative Review. Neuroscience & Biobehavioral Reviews, 105986.

Many thanks. This is a very helpful review paper. We have given a brief discussion of some of these mechanisms and cited this paper and the previous one in the following sentence added to that paragraph: "These hypotheses include the possibility that we make use of cues based on how sounds change under self-motion, audiomotor calibration and feedback from touching objects that produce sounds (Lewald 2013, Gori et al. 2014, Kolarik et al. 2025)". (line 20)

The manuscript assesses interesting variations of the learning procedures, including constraining the agent's rotational range to a small window, a monaural Teacher circuit without relying on the bilateral acoustic cues, and extending learning to both the azimuth and elevation dimensions. Unless I have missed it, has a variation also been discussed that accounts for calibration of auditory space necessitated by changes in acoustic information as the head grows bigger from infancy to adulthood, and do changes in head size factor into the models discussed (e.g. resulting ILD changes)? The manuscript points out in the abstract that "The acoustic cues used by humans and other animals to localise sounds are subtle, and change during and after development," and this issue has been discussed in the literature, therefore clarifying the answers to these questions in the text would be helpful.

We agree that the wording in the abstract in particular suggests we would be looking at changing head size during development. We have changed this wording to avoid making this suggestion. We have also added a sentence in the Results where we introduce the sensory disruptions making it clear that we do not discuss head size changes: "Note that that we do not address the much slower changes to acoustic cues during development, as head size changes." (line 234)

Lastly, the manuscript discusses how spatial maps of sound source azimuth and elevation might be calibrated and maintained, however distance is not prioritised (line 596 "Typically, sound source localization is simplified to determining the direction of arrival (DoA) of the sound, without estimating the source's distance from the listener"), and it is unclear why this is the case. Auditory distance is an important aspect of sound localization, and the issue of how this is calibrated (especially for blind individuals) is of current interest in the field. Perhaps some discussion of how the proposed models might help to calibrate auditory distance would make this aspect of the manuscript more complete.

We think it is likely that bootstrapping would help calibrate predictions of sound distance, however it would considerably complicate the model setup. Firstly, we would need to make use of some knowledge of sound source attributes (e.g. the typical sound level of a dog barking) and acoustic environment (echoes). We have added a note to the Limitations section of the discussion: "We only attempt to estimate direction of arrival and not distance." (line 552) And to the Methods: "To reduce the complexity of the model and data required, and in line with many other studies in the field, …" (line 620)

Minor comments
Fig 1. Caption: The information here needs to be a little clearer, as the labelling and clarity of the text could be improved and will be difficult to follow for a reader unfamiliar with acoustics. For example, the caption reads: "The auditory space map need to be learned and continuously re-calibrated during lifetime. (A) Different learning paradigms and the overall model of the Agent for spatial hearing. The acoustic environment is simulated using pre-recorded head-related transfer functions, converted a spatial sound stimuli into a cochleagram…" For more clarity, small suggested changes could include: "The auditory space map needs to be learned and continuously re-calibrated over the individual's lifetime. (A) Different learning paradigms and the overall model of the Agent for spatial hearing. The acoustic environment is simulated using pre-recorded head-related transfer functions (HRTFs), converting a spatial sound stimulus into a cochleagram…" The word cochleagram is mentioned in the caption and text, but unless I have missed it, it has not been defined. I would suggest explaining in a few words what this is to help the reader. Labels in the figure need explanations in the caption. I assume LF and HF refer to Low Frequency and High Frequency, but these need to be clarified. Similarly, does ILD refer to Interaural Time Difference here? This is mentioned later in the manuscript, but should be briefly mentioned in the caption when the reader is first introduced to it. DNN (deep neural network) should be specified in the caption so that the reader does not have to refer to the main text.

We have clarified these points in the new caption.

Fig 2: Some of the labels are very small and hard to read e.g. Small Teacher, Big student, or the small letters for L and R in panels B and C. I would suggest making them larger.
Fig3: Some of the light grey labelling makes it difficult to read (e.g. clockwise bias).
Fig 5: The labels SBC and GBC are very small.
Fig 6: The labels in panels f and h are very small.
Fig 8: Many of the grey labels within the plots (especially the numbers) are very small and hard to read.
Fig 9 caption: "Within each ear, a bandpass filter bank (ERB)…" Define ERB here.
Line 114: "gradient decent…" Typo? "gradient descent…"
ILD is defined twice on lines 120, and 130.

We have also made all the minor corrections listed above.

Reviewer #2

Spatial hearing has to be continually recalibrated due to differences in the geometry of the head and ears, particularly when these structures are growing, and when acoustic inputs are modified following hearing loss, etc. Because of its (normally) greater accuracy and reliability, many studies have focused on the role of vision in guiding auditory spatial processing, leading to the notion that a form of supervised learning takes place in the brain. While this is correct up to a point (principally because of evidence from studies of crossmodal plasticity in the superior colliculus during development), it is now well established that accurate sound localization can emerge without vision and that plasticity resulting from altered acoustic inputs is not dependent on (though may be enhanced by) concurrent visual information.

This study shows that a deep neural network can learn to localize sound without being provided with explicit teaching signals that might correspond to visual spatial cues and therefore stands apart from previous modeling studies in this area, which have been based on supervised learning. It also provides a potential framework for investigating the factors that give rise to the plasticity that has

been demonstrated in psychophysical and neurophysiological studies. As such, I think this paper could be an important contribution.

The notion of “bootstrap learning” to establish accurate sound localization using an innate brain function – for example, neonatal orienting movements enabling left-right discrimination – is plausible. There are, however, various places where the assumptions and results deviate from the biology, raising questions over the feasibility of the strategies proposed. Also some of the terminology used in the paper is misleading.

Throughout the paper, the authors describe the DNN’s estimate of sound source location as an auditory space map. While it may be expedient to do so for describing the model outcomes, the only region of the brain where a coarse *map* of space is constructed is the superior colliculus, where it is superimposed on to a map of visual space. The auditory system itself (including auditory cortex and inferior colliculus, IC) does not represent sound source location topographically, so I would recommend that the authors consider using alternative terminology (e.g. the network's representation or estimate of sound source location) to avoid misleading readers.

We agree. We were using ‘map’ in the sense from mathematics, but indeed this is likely to be misleading to a neuroscience audience. We changed throughout to use the word ‘localiser’.

Why was the lateral superior olive selected as a Teacher circuit? Interaural time differences are often claimed to be the dominant acoustic cue used by humans to localize sound, so some justification for why the LSO was selected rather than the medial superior olive would be helpful.

In general it is considered that ITDs are more useful at low frequencies where ILDs are small, and ILDs at high frequencies where ITD becomes ambiguous/noisy. For our models, we focused on ILD to keep the neural network simple. Representing fine temporal differences is not entirely straightforward in an artificial neural network, and would probably require a spiking neural network simulated with a high degree of temporal fidelity, which is computationally challenging to work with. We added the following to the results to acknowledge this: “Note that we chose to use only ILDs rather than interaural time differences (ITDs) to keep the model's neural network architecture simple and to avoid the use of spiking neural networks which are more challenging to train, although recent research attempts to address this issue.” (line 144)

The statement in second paragraph on page 2 (lines 27-38) about the accuracy of “innate neural circuits” needs more explanation (and references).

It is challenging to estimate the accuracy of the auditory orienting response, and although there is literature on this, we feel it would be a distraction from the main message of our paper which does not depend on this. We have therefore replaced this statement with the following: “However, an innate circuit is not sufficient as a localiser given that auditory cues change during a lifetime.” (line 36)

The term “spatial sound” is used in several places. The word “spatial” doesn’t add or mean anything informative (“spatialized” or “externalized” might be better but none of them are necessary in the contexts used).

We have removed the word “spatial” in this context throughout.

The absolute errors produced by the student network are very small and uniform across azimuth.

This is very different from real localization accuracy, which varies greatly with sound source location. This shortcoming should be discussed.

The errors are broadly comparable to human performance at midline and do increase towards the periphery, but indeed much less than those in humans. We have updated the text to reflect this: “The magnitude of errors at midline matches human behavioral studies well, although human performance at the periphery is much worse than the predictions by our model” (line 158). We also updated the figure in response to comments from reviewer 3 to make the pattern clearer.

Line 156: “single-LSO circuit”. Line 161: “single LSO”. What does that mean? One side of the brain?

We agree this wasn’t clear – it means a single LSO neuron. We have reworded it and other related terms like LSO-population to LSO neuron population.

A strength of the model is the capacity to manipulate the inputs to try to replicate different types of hearing loss. Although the effects of the manipulations shown in Figure 4 are interesting, I am not convinced that bilateral/unilateral scaling of ILD sensitivity accurately reflects loss of non-linear compression in the ear and therefore whether this is a useful transformation to incorporate in the model. The first paragraph on page 10 has no references to sport this. Unilateral shifting would have provided a closer match to the many experimental studies of unilateral conductive hearing loss.

The idea was to consider a set of transformations that capture some but not necessarily all of the components of hearing loss using a simple linear model that doesn’t make strong assumptions. We have rewritten the paragraph as follows: “These conditions simulate possible components of hearing loss or disrupted auditory signal compression across a broad frequency range. In reality, hearing changes often involve complex, frequency-dependent combinations of both shifting and scaling transformations, as well as nonlinear effects, which typically require detailed empirical measurements to characterize precisely. Our computational model enables the isolation and analysis of some of the abstract components of these transformations individually, allowing us to cleanly examine their effects.”(line 245) Indeed there are likely many nonlinear effects not captured by this, but this range of linear effects already has interesting characteristics. We added the text: “and we only consider linear effects in our model of hearing loss” to the limitations section of the Discussion.

Unilateral shifting is effectively already covered in the model since it would create a similar effect to asymmetrical scaling. We added the text: “We could also have considered asymmetrical shifting (e.g. L'=L-20,R'=R), however since this just introduces a bias in the Teacher, this would have a similar effect as asymmetrical scaling (fig 4 top right panel).” (line 241)

Incorporating limited plasticity in the LSO Teacher enabled recalibration of the student network following these manipulations but is there any evidence that this is biologically plausible? How might this be manifest in the LSO?

This is a good point. We refer to the teacher as LSO Teacher but in fact this function could be distributed at different points along the auditory pathway. We added the following to the Discussion: “We also do not attempt to locate a specific pathway or region of the brain in which the proposed learning takes place. For example, we talked about how the LSO Teacher model could incorporate limited plasticity, but this plasticity does not necessarily have to take place in the LSO but in any number of related sites along this pathway, for example in the inferior colliculus which is documented to exhibit plasticity (King et al. 1991, 2011, DeBello et al. 2004).” (line 564)

The model architecture is well designed. This included a stage in which the broadband sound was filtered by left and right ear HRTFs. How important is this step, particularly as the authors say that LSO neurons are not sensitive to these spectral cues? Could the spectral cues (a single HRTF) provide a teacher signal for calibrating binaural/azimuthal sensitivity (in the IC or at higher levels of the auditory pathway)? This possibility has been proposed in studies in which spectral cues have been manipulated.

It isn't essential to include HRTF filtering and indeed an earlier version of this project did not include it. Having a frequency-dependent pattern of ILDs is likely to make the task easier than having a single frequency-independent ILD. It is possible that our DNNs could be using monaural spectral cues, but we didn't test this. We do think that having a single HRTF as a Teacher signal is possible. One example is the "unilateral teacher" from Figs 6 and 7, but there may be other ways too. We highlighted this in the text by adding: "We next test a monaural Teacher circuit without relying on the bilateral acoustic cues but instead using simplified monaural spectral cues." (line 392)

The authors suggest that one source of "intrinsic reward" signal might be neurons tuned to midline sound sources (zero ILD), stating that this is consistent with "observed spatial tuning curves in the IC" [32-35] (lines 333-334). Only one of these 4 cited papers is about the IC and even then the statement is incorrect. The great majority of IC neurons have contralateral preferences and almost none are tuned to the midline. The modeling therefore predicts a feature that is shown by very few neurons, casting doubt over its feasibility.

We have modified the text to read: "observed spatial tuning curves along the auditory pathway" (line 347). Two of the four papers cited include data from IC, and two from auditory cortex. For our model, we do not require the majority of cells to have midline preference, only that some do. In fact, these cells are not so rare, for example in Uragun and Rajan (2013), in figure 7 we see that clusters 4-6 are all of this type, comprising 71 of 208 units they recorded in the central nucleus of the rat IC (and it is particularly clear in cluster 6 comprising 35 units).

The terms 2D and 3D space are used too loosely. The directional coordinates of a sound source are defined by the two dimensions of azimuth and elevation, whereas distance provides the third dimension. Very reasonably, distance is fixed to simply the modeling but using 2D and 3D space to refer to azimuth and azimuth plus elevation, respectively, is misleading.

We agree and we have updated this terminology throughout. We refer to "circular localiser" or "spherical localiser" for what we referred to previously as "2D map" and "3D map".

There are a few typos in the manuscript. These include but are limited to:
Lin 199: "due the"
Line 598: "model is simplify to be"
Line 781: "sphereical"

We have fixed these.

Reviewer #3 (pasted from PDF)

Summary:

The authors address the problem of how accurate auditory spatial maps—specifically for broadband white noise stimuli—can be learned from coarse-grained supervisory signals. They investigate the hypothesis that innate "teacher" circuits in the brain may provide minimal feedback signals that guide the learning of "student" circuits through a process termed bootstrap learning. The paper

explores several biologically plausible mechanisms by which such teacher circuits might be instantiated, including left/right comparators, midline detectors, and temporal comparators. The authors demonstrate that student networks can learn precise spatial maps, adapt to disruptions in the teacher circuit, and succeed regardless of the specific implementation of the teacher network.

Main review:

The motivation of the paper is clear, and the general problem is of broad interest. The core idea presented in the paper is simple, neat, and has the potential to be expanded more generally.

Having said that, this manuscript can benefit from several clarifications and improvement before being considered for publication. I'm listing my concerns below, and I hope that the authors can clarify them.

1. How are the authors defining "calibration": In several places in the manuscript the concept of "calibration" is referenced without proper definition. Are they referring to the statistical notion of calibration in the sense that uncertainty (or predicted error) in estimation of new observations inversely mirrors accuracy? It seems like here calibration refers to a process akin to fine-tuning. It would be good to define this term operationally since its used in several places in the text.

We added the definition in the introduction "calibrated (that is, adjusted from an approximately correct starting point, or fine-tuned)"(line 11). Fine-tuned is the closest alternative phrase but we felt that it is not one that would necessarily be clear to some readers from a more experimental neuroscience background.

a. L6: "adjusting to developmental changes like head growth": This is a tricky example as its not clear if accuracy is held constant while these changes happen.

While it may not be constant, it certainly must adjust since 5-year old children are able to match the localisation accuracy of adults (Van Deun et al. 2009). We have added this citation.

2. Independence of Teacher and Student modules: Conceptually, in this approach the authors suppose the independence of these two components. In other words, one can swap out different teacher modules, such as the LSO circuitry for other mechanistic implementations, and re-learn the spatial map. But this seems to be a strong constraint in the sense that in biological systems brainstem circuits like the LSO and MSO are early, upstream components of the auditory pathways used for sound localization. This, I believe, will have consequences for the learning process.

We agree things are likely not so clear-cut in the brain. We added the following to the Discussion. "We abstracted the Teacher and Student into separate independent modules, but in real brains it is unlikely that there is such a clear-cut distinction. Instead, our model should be considered at the behavioral level rather than at the implementation level." (line 562)

3. System identification under variable source spectra: A fundamental challenge in sound localization is that listeners typically have no access to the source spectrum, which complicates the inference of spatial location from sensory input. This issue is particularly pronounced for localization in elevation, where spectral cues are more ambiguous. While the paper focuses primarily on azimuthal localization, it remains unclear how the proposed bootstrapping process would perform—either theoretically or empirically—when the teacher circuit's response does not uniquely map to location (i.e., the response function of the teacher isn't one-one). This ambiguity could undermine the reliability of the supervisory signal and seems critical to address. I recommend the authors provide

additional theoretical justification or empirical evidence to support the viability of the approach under such conditions.

There are two potential issues here. The first is the extent to which our model could generalise to the case of a sound with an unknown source spectrum. In principle, a sufficiently large DNN will be able to learn this (because it can also learn to recognise the sound source even if we only give feedback on sound location). However, when we investigated this practically we found that it required a much larger DNN and more training data, pushing it beyond the reach of our available computing resources. The second issue is whether or not a non-1-to-1 Teacher response function compromises learning. To this question the answer is no, since the Teacher only uses ILD which is already ambiguous, while the Student is nonetheless able to learn and resolve the ambiguity.

4. LSO teacher implementation: The performance reported in Fig. 2G doesn't match the authors characterization of "lowest error near the midline and increased errors at ±90". Can the authors replot the these graphs only for this range of azimuths and provide some statistics to back this claim? I'm also unclear on how I must interpret the significant non-monotonicity in these charts.

We agree that on further reflection our data are not sufficient to support this claim, so we have removed it. In terms of the 'significant non-monotonicity', we do see something similar in human data (Carlile et al. 1997 "The nature and distribution of errors in sound localization by human listeners" Fig 9), although it may also be an artefact of the HRTF recordings or model setup which does not change our overall conclusions.

a. I'm assuming these errors are single-shot predictions during the test phase. Is this correct?

This is correct.

b. Can the authors also provide some quantification for how error varies with the number of head movements the agent performs? In other words, in one-shot the agent can estimate if it overshoots or undershoots the target. But what's the sequence of actions that follow after? Do the authors cap the movement to n=2 (as suggested by Algorithm 1)?

We are not modelling how error improves after a series of head movements, only the learning signal that it provides. We would need a different model if the aim was to use head movements to minimise within-trial accuracy.

5. Midline detector as intrinsic reward: Fig. 5 gives the impression that the reward is +1 for the midline and -1 otherwise. While I can understand how this would work if the student has access to the firing rate of the teacher (as it has a smooth drop off on either side that can provide gradient information), it's unclear to me how this binary reward function helps learning. Does the student rely on random exploration of lateral movements until it happens upon the midline? If so, this seems inefficient and potentially implausible. Can the authors clarify?

The logic of the model here is that the actual real-world reward *is* sparse, stochastic and binary (+1 or -1), which is very inefficient but it's the conditions that we have to learn to deal with. We mention in the text: "external rewards—such as successful prey capture—are typically sparse in real-world environments". Reinforcement learning (RL) algorithms are sometimes able to learn despite these very sparse rewards. Here we propose an additional element to improve the efficiency of the RL algorithm. We use the midline detector as a sort of internally generated reward that is present on every trial. To make it clear: we are not considering a case where the model gets a large number of guesses at the location and has to keep turning their head until they get the reward. Instead, it gets only a single try to rotate toward the midline to get this stochastic binary reward.

a. On the other hand, Fig. 5D (error vs. azimuth) looks quite human-like.

We agree, but we do not want to make a strong claim about this.

6. Adaptation vs degradation: The authors repeatedly emphasize that their approach supports adaptation when aspects of innate function—such as teacher responses—change. However, this could be conceptually distinct from scenarios involving impaired hearing or reduced acuity. In such cases, the “teacher” circuit may remain intact, while the “student” circuit exhibits high localization errors. It is unclear how the proposed bootstrapping mechanism would handle this type of asymmetry. I would appreciate further clarification or intuition in this regard.

The case when the teacher is intact but the student has high errors is the initialisation stage for the first models. The later models of hearing impairment cover the harder case when both the student and teacher are impaired (since we disrupt the *signal* not the teacher circuit).

7. The authors don’t make explicit how they theorize the multiple mechanisms to interact with each other as suggested in the abstract.

We did not mean to imply that we had a general theory of how multiple mechanisms interact, but in Figs 6 and 7 we provide examples of this. We changed the abstract to read: “We find several possible neural mechanisms that could underlie this type of learning, and hypothesise that multiple mechanisms may be present and provide examples in which these mechanisms can interact with each other.”

8. Grammatical errors: There are several places in the manuscript that can benefit from proofreading and editing. I’m listing a few (not exhaustive) below but please do ensure a thorough review.

a. Abstract: “or other supervisory signal”.

b. L92 “ The agent uses Student network”

c. L97 “listen again”

d. L109 “that for a”

e. Fig 1 caption “map need to”

f. Fig 6 heading “unllateral”

We fixed the errors (except f which is correct but may render strangely at some zoom levels).

9. L423 and L547: sound source localization (SSL) – This acronym isn’t used anywhere else in the text and it also bears the risk of being confused with the more popular “self supervised learning” usage in machine learning.

We removed this acronym.
